# How Socially Avoidant Emerging Adults Process Social Feedback during Human-to-Human Interaction after Social Rejection: An Event-Related Potential Study

**DOI:** 10.3390/bs14060457

**Published:** 2024-05-29

**Authors:** Yangdi Chen, Xinmei Deng

**Affiliations:** 1 School of Psychology, Shenzhen University, Shenzhen 518060, China; 2 The Shenzhen Humanities & Social Sciences Key Research Bases of the Center for Mental Health, Shenzhen University, Shenzhen 518060, China

**Keywords:** social avoidance, social rejection, emerging adults, interpersonal interaction, event-related potentials (ERPs)

## Abstract

Social avoidance refers to active non-participation in social activities, which is detrimental to healthy interpersonal interaction for emerging adults. Social rejection is a kind of negative social evaluation from others making people feel social pain. However, how socially avoidant emerging adults process social feedback information after experiencing social rejection has received less attention. The current study aimed to explore the differences in social interaction feedback processing after social rejection between a socially avoidant group (*n* = 16) and a comparison group (*n* = 16) in a human-to-human interaction context. Computer game tasks with two types of interaction (cooperation and competition) were used to record the event-related potentials when receiving social interaction feedback in two conditions (social rejection and control condition). The results showed that (1) the socially avoidant group had lower reward positivity amplitudes than the comparison group when receiving social feedback; (2) the socially avoidant group presented larger P300 amplitudes in the social rejection condition than in the control condition, but the comparison group did not; and (3) social rejection evoked more negative N1 amplitudes in the socially avoidant and comparison groups. The findings suggest that socially avoidant emerging adults may have flaws in reward sensitivity during interpersonal interaction, and they might also exert more attentional and emotional resources to social feedback after social rejection.

## 1. Introduction

Positive interpersonal interaction is crucial for emerging adults to foster a sense of social connectedness and promote social adjustment [1]. However, social avoidance, an unwillingness to be involved in social activities and interpersonal interaction, impedes their adaptive development [2]. According to several previous studies, socially avoidant individuals struggle in their emerging adulthood with internalizing problems (e.g., anxiety, depressive symptoms) and poor relationships [3,4]. They usually have negative self-images and negative beliefs about interpersonal relationships [5]. As a result, socially avoidant individuals engage in avoidance behavior in social contexts to keep away from distressing social outcomes [6]. When they are exposed to a negative social context, it would be worse. Research has shown that negative social situations (e.g., rejection, exclusion) are perceived as threats by socially avoidant individuals, leading to fewer positive emotions, more negative emotions, and fewer cognitive resources to cope with interpersonal interaction [7,8]. Social rejection is a negative evaluation from other people in social situations, which is challenging to socially avoidant individuals and undermines their desire to interact with others [9]. However, little is known about socially avoidant emerging adults during interpersonal interaction when undergoing social rejection. In recent years, event-related potential (ERP) has enabled the measurement of the neural underpinnings of social cognition [10]. Therefore, the present study aimed to examine differences in cognitive processes between socially avoidant and comparison emerging adults during actual human-to-human interpersonal interaction after undergoing social rejection. This can facilitate our understanding of emerging adults with social avoidance.

### 1.1. Social Avoidance and Interpersonal Interaction

Social avoidance is a subtype of social withdrawal, characterized by high avoidance and low approach motivations during social interaction [11]. In the collective, socially avoidant individuals prefer to be alone and actively avoid social interaction [12]. Compared with non-socially avoidant individuals, they are less responsive to peers during interaction [4]. One possible explanation for the avoidance is that they avoid social risks such as being negatively judged by their peers [13]. In other words, socially avoidant individuals might actually care about others’ evaluation, even though they avoid interacting with others.

Prior ERP studies have provided findings related to social avoidance. Perry et al. found that individuals who tended to keep a greater distance from strangers had less negative N1 amplitudes in a virtual social situation requiring participants to determine a comfortable interpersonal distance [14]. It suggests that socially avoidant individuals may exert fewer early attentional resources to social information, in order to minimize the discomfort of interacting with others. However, in an emotion recognition task, Cui et al. demonstrated that people who are afraid of being judged by others had more negative N170 amplitudes, which suggests that socially avoidant individuals may exert more early attentional resources to socio-emotional expressions [15]. The conflicting evidence suggests the necessity of further research on social avoidance. In addition, reward positivity (RewP) is thought to be correlated with social approach and avoidance motivations [16], but the evidence is still insufficient. Most importantly, there are few ERP studies that explore social avoidance in a real social situation.

An interpersonal interaction setting is conducive to examining the behaviors and cognitive processes of socially avoidant individuals [17]. According to Kashdan et al., those who relied on social avoidance strategies in interpersonal interaction with strangers reported a high level of social anxiety due to the fear of negative evaluation and rejection [18]. Previous research has shown that, when dealing with necessary interpersonal interaction, socially avoidant emerging adults would feel stressed and have poor-quality relationships with others [3]. After that, they are probably unable to receive positive social evaluations from their peers, and their accumulated negative social evaluation would increase their social avoidance motivation [19]. Considering that socially avoidant emerging adults show intense social avoidance during interpersonal interaction, real interpersonal interaction would be an ideal situation to examine the ERPs related to social avoidance.

Cooperation and competition are two commonly used and opposite models of interpersonal interaction, both of which require predicting others’ intentions and adjusting one’s own action accordingly [20]. Thus, cooperation and competition are considered good interaction examples of social and emotional sharing [21]. Additionally, many studies have shown that the neural responses during cooperation and competition are different. For example, Tsoi et al. demonstrated that brain activation of the left TPJ (temporoparietal junction) was higher in competition than cooperation, most likely because of the different social motivations [22]. The high avoidance and low approach motivations of socially avoidant individuals may be expressed in cooperative and competitive tasks [11]. Most importantly, it would be difficult and uncomfortable for socially avoidant individuals to engage in tasks without specified goals (e.g., face-to-face talking interaction) [23]. Cooperative and competitive tasks with specified goals may make it easy for socially avoidant individuals to interact with others, especially strangers in the present study. Thus, examining brain activity across cooperative and competitive interaction helps to understand the different patterns of interpersonal interaction in socially avoidant emerging adults.

### 1.2. Social Rejection

Social rejection is a kind of negative social evaluation that makes people feel hurt, depressed, or even angry, and is considered an undesirable treatment in interaction [24]. An online experiment led participants to believe they were playing a virtual tossing game with two other people and fewer received throws in the game indicated less acceptance from the other two players [25]. It demonstrated that remote and artificial social rejection via the Internet could also make participants feel bad. Previous ERP research has revealed that social rejection can be quickly perceived with specific components in the frontal area (e.g., N1, P300), reflecting a related emotion and cognition process [26]. Pegg et al. also found that social rejection could elicit prominent N1 and P300 amplitudes, suggesting that people might mobilize high attentional and emotional resources to rejection [27]. According to the multimotive model, social rejection may elicit people’s unintentionally social facilitative actions to gain acceptance from others [28]. People may think of rejection as meaningful social information. However, it is still unclear whether socially avoidant individuals care about other’s acceptance after experiencing social rejection, particularly among socially avoidant emerging adults [29,30]. In the present study, social rejection was experimentally induced to make participants feel rejected and the interpersonal interaction was person-to-person in a real setting.

### 1.3. ERP and Social Feedback

The neural responses to social feedback are often used to reflect the cognitive and emotional process [27,31,32]. Event-related potential (ERP) is considered a feasible tool to examine the neural underpinnings of social feedback processing depending on its high temporal resolution [33]. Based on previous research, there are several ERP components reflecting feedback processing, such as N1, feedback-related negativity (FRN), P300, and RewP [27,34]. 

N1, an early negativity, peaks around 150 ms after feedback onset at frontal sites, indicating attention to feedback [35]. FRN, a negativity, peaks around 250 ms after feedback onset at frontocentral sites, indicating the unexpectation and displeasure to negative feedback [34]. P300, a positivity, occurs around 300 ms after feedback onset in multiple brain regions, such as prefrontal, frontal, and parietal, reflecting emotional attention during feedback processing [36]. RewP, a kind of difference wave calculated as positive feedback potential minus negative feedback potential, occurs around 300 ms after feedback onset at frontocentral sites, reflecting reliable responsiveness for positive social feedback [32]. In contrast to subjective perception assessment, real-time ERP measurements for social feedback processing may provide more accurate and objective data on socially avoidant individuals in emerging adults [37].

### 1.4. Present Study

Coordinated interpersonal interaction is conducive to emerging adults’ mental health and development in society [38]. However, social avoidance hampers proper social interaction, which has been shown to be a serious threat to emerging adults’ well-being [3]. Many studies have focused on social rejection in the general population, showing that rejected individuals feel bad and subsequently hope to regain acceptance [39,40]. Additionally, neuroscience provides relevant evidence that social rejection is correlated with the activity of some frontal brain regions (e.g., right ventral prefrontal cortex (RVPFC)) [41]. Nevertheless, few neuroscience studies have employed real human-to-human interaction to conduct research on socially avoidant emerging adults in a social rejection condition. Therefore, by using the ERP technique, the present study aimed to examine how socially avoidant emerging adults process social feedback during real interpersonal interaction after undergoing social rejection, compared with comparison emerging adults.

According to previous studies, we hypothesized that the social rejection condition would evoke more negative N1 amplitudes than the control condition in socially avoidant and comparison groups (Hypothesis 1) due to their need to restore a sense of belonging and nonconscious expectation to gain acceptance [28]. Next, failure feedback would evoke more negative FRN amplitudes than success feedback in socially avoidant and comparison groups (Hypothesis 2) due to the negative expectation of failure feedback [36]. In addition, the social rejection condition would evoke greater P300 amplitudes than the control condition in socially avoidant and comparison groups (Hypothesis 3) as a result of their enhanced negative emotion from rejection [26]. As for RewP, the socially avoidant group would have lower amplitudes than the comparison group (Hypothesis 4) due to their low social approach motivation and reward sensitivity [11,16].

## 2. Materials and Methods

### 2.1. Participants

We used G*Power 3.1 software to calculate the required sample size [42]. According to the 2 (Group: socially avoidant group vs. comparison group) × 2 (Condition: social rejection vs. control) × 2 (Task: cooperation vs. competition) × 2 (Feedback: success vs. failure) repeated measures mixed-design adopted in the current study, we used F tests (ANOVA: Repeated measures, within–between interaction) to conduct the priori power analysis. The parameters were set as follows: effect size = 0.25, confidence level α = 0.05, number of groups = 2, and number of measurements = 8. To achieve the power factor of 0.90, at least 20 participants were demanded in total. Given that RewP is a kind of difference wave with a 2 (Group: socially avoidant group vs. comparison group) × 2 (Condition: social rejection vs. control) × 2 (Task: cooperation vs. competition) repeated measures mixed-design (without Feedback factor), we set the number of measurements as 4 to calculate the required sample size again. At least 30 participants were demanded in total. To prevent the impact of unexpected bad electroencephalography (EEG) data, we invited 34 emerging adults (17 for the socially avoidant group and 17 for the comparison group) from a large sample of 430 voluntary college students (161 males, 269 females; 17–26 years of age, *M*_age_ = 19.46 years, *SD* = 1.71) recruited through flyers based on their self-reported social avoidance scores (see details in Measures). The large sample was recruited through flyers on a voluntary basis. Two emerging adults of the same sex, respectively, from the socially avoidant group and comparison group were strangers and were randomly matched as a male–male or female–female dyad to complete the experiment of interpersonal interaction together. 

Two female participants were excluded from the final samples on account of the bad EEG data (the percentage of valid trials was lower than 80%). Therefore, the final participants included 16 individuals in the socially avoidant group (4 males, 12 females; *M*_age_ = 19.81 years, *SD* = 1.42) and 16 individuals in the comparison group (4 males, 12 females; *M*_age_ = 19.69 years, *SD* = 1.08), in accordance with the sample size of typical ERP studies [43]. The analyzed sample (*n* = 32) was not significantly different from the large sample in terms of descriptive characteristics (Gender: *t*(459) = −1.543, *p* = 0.131, *d* = −0.28. Age: *t*(459) = −1.217, *p* = 0.231, *d* = −0.19).

All participants were right-handed, had normal or corrected-to-normal eyesight, and did not report any psychological abnormalities. They all provided informed consent before the experiment. This study, approved by the Institutional Review Board of Shenzhen University (PN-202300065), was conducted in accordance with the ethical standards formulated in the 1964 Declaration of Helsinki.

### 2.2. Measures

We used the Chinese version of the Social Preference Scale––Revised (SPS-R, α = 0.85) to assess emerging adults’ social avoidance [44]. The 5-point (1 = *Not at all* to 5 = *A lot*) scaled SPS-R has four subscales: shyness (e.g., “Even though I want to be with others, I would feel nervous about interacting with them.”), unsociability (e.g., “I don’t have a strong need to be with others.”), social avoidance (e.g., “I prefer to be alone rather than with others.”), and isolation (e.g., “I want to be with others but they often don’t want to be with me.”).

Based on substantial studies, shyness and unsociability have been found to be highly correlated with the degree of social avoidance [44]. Thus, we screened the two groups of participants based on the 75% criterion, which is a classic approach in social avoidance research and has undergone rigorous validation [45]. Specifically, the socially avoidant group (*n* = 17) came from the upper 25% for social avoidance scores but were in the bottom 75% for shyness and unsociability scores of the original large sample. The comparison group (*n* = 17) came from the bottom 75% for social avoidance, shyness, and unsociability scores of the original large sample. The difference in the total social avoidance scores between the two groups was significant (*M*_avoidant_ = 23.29, *SD* = 2.66; *M*_comparison_ = 15.88, *SD* = 2.93; *t*(32) = 7.711, *p* < 0.001, *d* = 2.65).

### 2.3. Procedure

Participant dyads of the same sex (a dyad = a socially avoidant and a comparison individual) were asked to complete two experimental conditions in counterbalanced order: (a) the social rejection condition, and (b) the control condition (without social rejection). The study required two visits to the lab. To diminish the impact of the first visit, the dyads were asked to visit the lab for the second time after one week. On the first visit, a general introduction about the procedure of the experiment was given, and then participants signed the informed consent forms and completed self-report questionnaires about general demographics.

**Figure 1:**
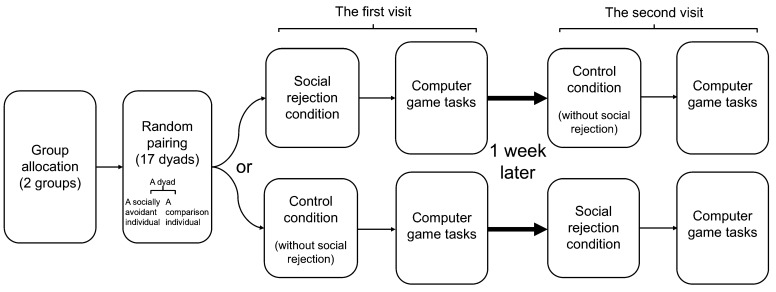
Schematic illustration of the procedure.

In the social rejection condition, participant dyads were required to sit next to each other and complete self-report questionnaires about relevant psychological variables (see Appendix B). Meanwhile, the EEG was attached to the two participants by research assistants. Then, two social rejection paradigms (see details below) were conducted to elicit participants’ feelings of being socially rejected. Subsequently, the dyads were required to complete the computer game tasks (see details below), and in the control condition, the dyads sat next to each other and completed the same questionnaires to the social rejection condition during EEG placement. After that, the dyads completed the same computer game tasks to the social rejection condition. The overview of the procedure is illustrated in Figure 1.

#### 2.3.1. Social Rejection Paradigms

According to the responsive theory of social exclusion, there are three options to experimentally induce social rejection: explicit rejection (clearly stating no), ostracism (ignoring), and ambiguous rejection (being unclear) [46]. Thus, two social rejection paradigms were employed successively to experimentally induce the participants’ robust and comprehensive feelings of being socially rejected. in particular, the social evaluation task was used to induce explicit rejection and the “social rejection” picture processing task was used to induce ostracism. 

Initially, it was a social evaluation task [47]. It was explained to participants that they were taking part in a study on how individuals form initial impressions. They were asked to provide their ID photos for the study. We made them think that some peer strangers in the university had rated their images. On the visit of the social rejection condition in this study, the participants had access to those ratings on the computer screen. In fact, all ratings from strangers were generated pseudorandomly. A total of 47 strangers (94%) gave the participant a negative social evaluation and 3 strangers (6%) gave a positive social evaluation. According to typical research on social rejection, the predetermined percentage is used to elicit the participant’s sense of being socially rejected with higher validity [48].

Then, a “social rejection” picture processing task was performed. Forty “social rejection” images were chosen from the “social exclusion” category of the ISIEA, an image database of social inclusion and exclusion in young Asian adults [49]. Each of the 40 images depicted a scene in which one rejectee was physically and facially expressing sadness or frustration, while others (rejecters) were laughing and talking together. During the task, the dyads were asked to view the 40 pictures as though they were the rejectees in the scene. After viewing each picture, they pressed keys to indicate the valence (1 = *sad* to 5 = *happy*) and intensity (1 = *calm* to 5 = *strong*) of their emotions. 

#### 2.3.2. Computer Game Tasks

**Figure 2:**
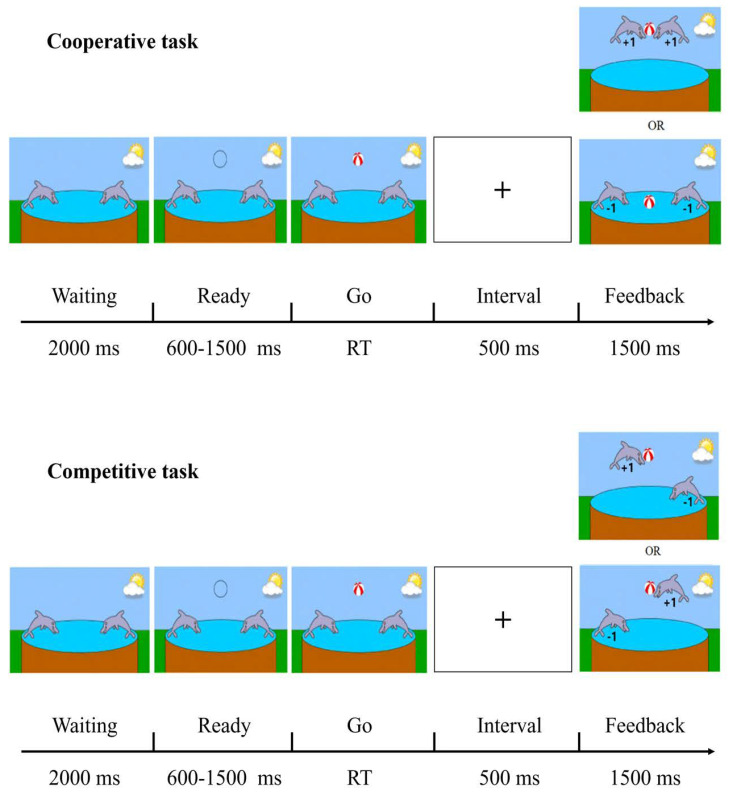
Schematic illustration of cooperative and competitive computer game tasks.

The computer game tasks we employed were designed by Cui et al. [50] and include a cooperative task and a competitive task with random order. There were 50 trials each for the cooperative and competitive tasks. Each trial began with two dolphins on the screen. The left participant controlled the left dolphin and the right participant controlled the right dolphin. A total of 2000 ms later, a hollow circle appeared to signal the dyads to get ready. After a random interval of 600–1500 ms, the circle turned to a red and white ball indicating that participants could start to press the key to catch the ball. The left participant was instructed to press the “1” key on the left keyboard and the right participant was instructed to press the “0” key on the right keyboard to react. In the cooperative task, they were required to react as simultaneously as possible to catch the ball together. In the competitive task, they were required to react as quickly as possible to catch the ball first. Following the participants’ reaction, there was an interval of 500 ms, and then the trial feedback appeared on the screen and remained for 1500 ms at the end of the trial (Figure 2). All feedback in the task trials was genuine and was generated instantly based on participants’ reaction times.

EEG was measured during the computer game tasks, in which participants were required to minimize their head and body movements and refrain from speaking. Research assistants confirmed that the participants were clear about the rules of the computer game tasks. Before the formal trials, participants had access to five practice trials. Invalid trials in the computer game tasks caused by missing behavioral data were removed. On average, there were 47.18 (94.36%) valid trials for the cooperative task and 44.76 (89.52%) valid trials for the competitive task in the control condition, and 46.76 (93.52%) valid trials for the cooperative task and 43.76 (93.52%) valid trials for the competitive task in the social rejection condition. 

In the cooperative task, if the difference in reaction times (RTs) between the two participants (RT1, RT2) was below a threshold set to T = 1/8 (RT1 + RT2), the feedback would show that the two dolphins caught the ball together. In this case, the two participants cooperated successfully. They would receive success feedback and gain one point, respectively. Conversely, if the difference between RT1 and RT2 was above the threshold T, the feedback would show that no dolphin caught the ball. It meant the failure of the cooperation task. They would receive failure feedback and lose one point, respectively (Figure 2). The aim of the two participants in the cooperative task was to gain as many points as possible by adjusting their reactions to synchronize consciously. In the competitive task, the dolphin controlled by the participant who reacted faster would catch the ball. The faster participant would receive success feedback and gain one point. Accordingly, the slower participant would receive failure feedback and lose one point (Figure 2). The aim of the two participants in the competitive task was to gain as many points as possible by reacting quickly. To motivate the participants to actively join in the task, the participants were told that the points they gained from the task could be converted into monetary rewards after the study.

### 2.4. EEG Recording and Data Analysis

Throughout the computer game tasks, the EEG of the two participants was recorded continuously. A 64-channel portable EEG device (BrainAmp, Brain Products, Gilching, Germany) was used to collect EEG data, sampling at 500 Hz on the international 10/20 system. Two electrodes (TP9 and TP10) were placed on the left and right mastoids. The left mastoid (TP9) of the participant served as the online reference. The electrode impedance was kept below 10 kΩ. 

EEG data were analyzed by using BrainVision Analyzer 2.2 software. Data were re-referenced and were filtered with a pass band of 0.1–40 Hz. Onsets were set as the beginning of the feedback in the computer game tasks. EEG data were then segmented into 2500 ms epochs that extended from 1000 ms before the onset to 1500 ms after the onset. Independent component analysis (ICA) rejection was used to get rid of ocular artifacts, and manual inspection was used to remove other strong movement artifacts. Trials with artifacts over ±80 μV were then eliminated from subsequent analysis. On average, there were 47.81 (95.62%) valid trials for the cooperative task and 42.56 (85.12%) valid trials for the competitive task in the control condition, and 47.72 (95.44%) valid trials for the cooperative task and 40.53 (81.06%) valid trials for the competitive task in the social rejection condition. EEG data were then segmented into 4 categories: cooperative success, cooperative failure, competitive success, and competitive failure in 2 conditions of social rejection and control. In this case, there were 8 categories in total. Then the categorized EEG data were further baseline-corrected (200 ms before the feedback onset as baseline) and were averaged over all trials of each participant. Finally, the ERPs of the above-mentioned 8 categories were grand-averaged separately for the socially avoidant group and the comparison group.

Based on previous studies and a visual inspection, in this study, the N1 component was analyzed as the mean voltage between 100 and 170 ms after feedback onset at channel Fz [35]. The FRN component was analyzed as the mean voltage between 210 and 280 ms after feedback onset at channel Fz [31]. The P300 component was analyzed as the mean voltage between 300 and 410 ms after feedback onset at channel Fz [51]. The RewP component was analyzed as the mean voltage of success feedback minus the mean voltage of failure feedback between 250 and 350 ms after feedback onset at channel Cz [52]. 

As for neural data, the dependent variable is the grand-averaged amplitudes of each analyzed ERP component. Two (Group: socially avoidant group vs. comparison group) × 2 (Condition: social rejection vs. control) × 2 (Task: cooperation vs. competition) × 2 (Feedback: success vs. failure) repeated measures ANOVAs were applied, respectively, for N1, FRN, and P300. For RewP, we employed a 2 (Group: socially avoidant group vs. comparison group) × 2 (Condition: social rejection vs. control) × 2 (Task: cooperation vs. competition) repeated measures ANOVA, given that the RewP is a kind of difference wave mentioned above without the Feedback measurement factor. The behavioral and neural data were statistically analyzed by using SPSS 24.0. The predetermined significance level was *p* < 0.05. The Bonferroni test was used for post hoc tests.

## 3. Results

To make an operational check, paired sample *t*-tests were conducted between different experimental conditions. The results revealed that participants in the rejection condition reported lower positive emotion (*t*(31) = 3.605, *p* = 0.001), higher negative emotion (*t*(31) = −1.817, *p* = 0.079), and lower basic psychological needs (*t*(31) = 4.726, *p* < 0.001) than in the control condition without social rejection. Social rejection was effectively induced.

The results of behavioral data (RTs and successful rate) recorded during the computer game tasks are in Appendix A and Table A1. The neural results recorded during the computer game tasks are as follows.

### 3.1. N1 (100–170 ms, Fz)

There was a significant main effect of Condition, *F*(1, 30) = 6.66, *p* = 0.015, η_p_^2^ = 0.18. Compared with the control condition, social rejection evoked more negative N1 amplitudes. There was a significant main effect of Task, *F*(1, 30) = 21.37, *p* < 0.001, η_p_^2^ = 0.42. The competitive task evoked more negative N1 amplitudes than the cooperative task. There was a significant main effect of Feedback *F*(1, 30) = 8.86, *p* = 0.006, η_p_^2^ = 0.23. The success feedback evoked more negative N1 amplitudes than failure feedback. The main effect of Group was not significant, *F*(1, 30) = 0.08, *p* = 0.779, η_p_^2^ < 0.01.

**Figure 3:**
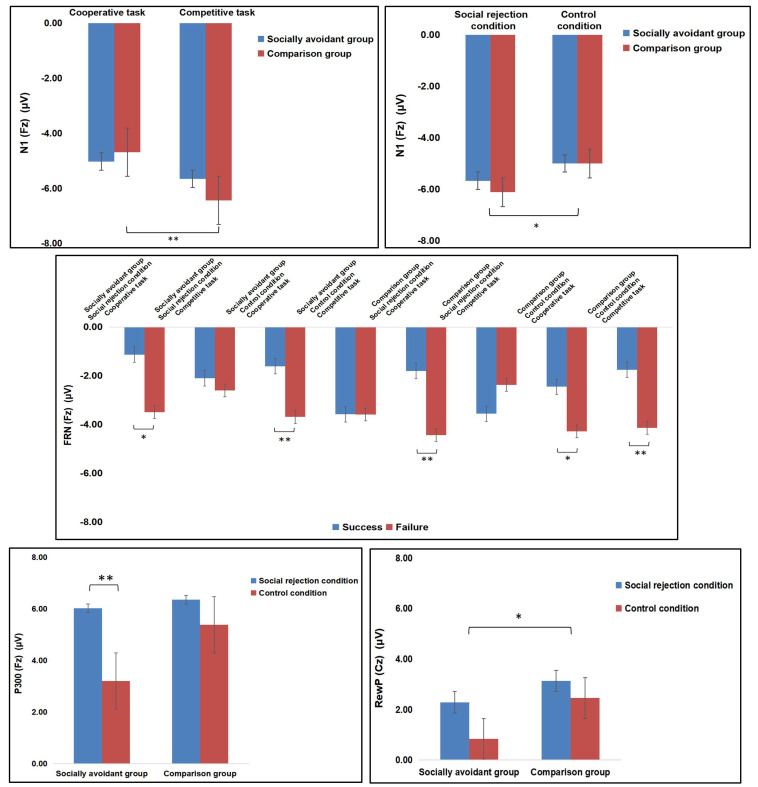
Grand-averaged amplitudes of N1, FRN, P300, and RewP in different conditions between socially avoidant group and comparison group. * *p* < 0.05, ** *p* < 0.01.

**Figure 4:**
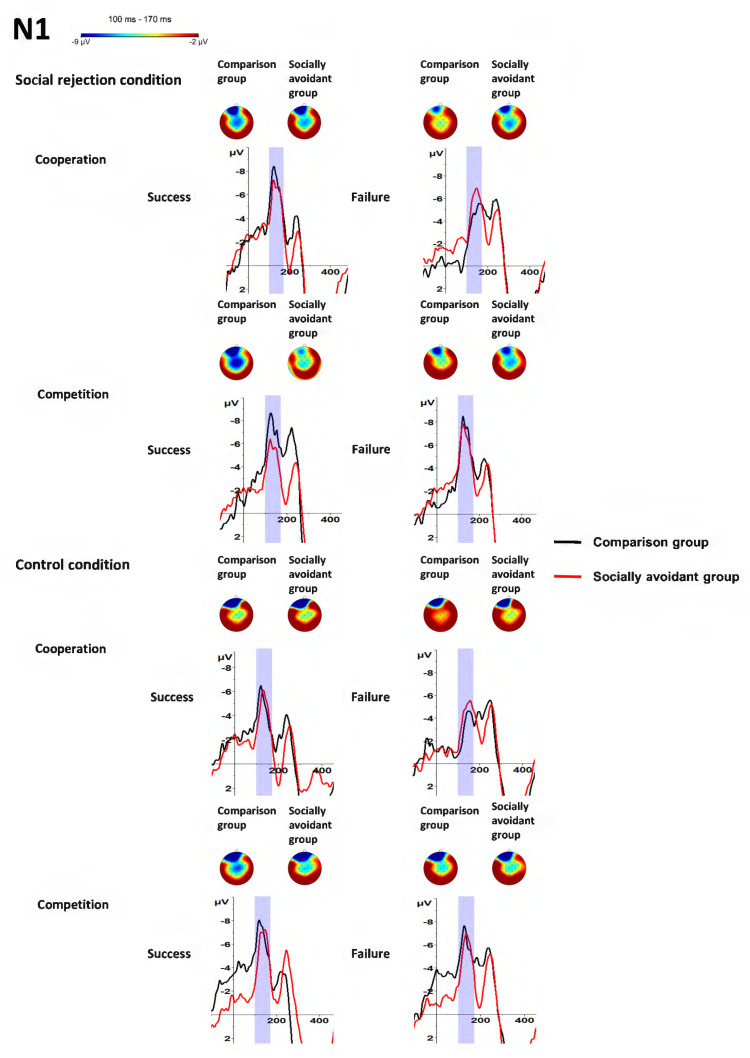
Grand-averaged N1 waveforms and scalp distributions in response to success and failure feedback in cooperative and competitive tasks in different conditions between socially avoidant group and comparison group.

The interaction of Group × Task was significant, *F*(1, 30) = 4.76, *p* = 0.037, η_p_^2^ = 0.14. Post hoc tests showed that competition evoked more negative N1 amplitudes than cooperation in the comparison group (*p* < 0.001) instead of the socially avoidant group (*p* = 0.095). However, the N1 amplitudes had no significant group difference in the cooperation (*p* = 0.670) and competition tasks (*p* = 0.370). The interactions of Task × Feedback were significant, *F*(1, 30) = 4.26, *p* = 0.048, η_p_^2^ = 0.12. Post hoc tests showed that success feedback evoked more negative N1 amplitudes than failure feedback in the cooperation (*p* = 0.001) instead of the competition task (*p* = 0.303), and competition evoked more negative N1 amplitudes than cooperation both for the success (*p* = 0.014) and failure feedback (*p* < 0.001). No other interaction was observed. The interaction of Group × Condition was not significant, *F*(1, 30) = 0.41, *p* = 0.529, η_p_^2^ = 0.01. The interaction of Group × Feedback was not significant, *F*(1, 30) = 2.65, *p* = 0.114, η_p_^2^ = 0.08. The interaction of Condition × Task was not significant, *F*(1, 30) = 4.03, *p* = 0.054, η_p_^2^ = 0.12. The interaction of Condition × Feedback was not significant, *F*(1, 30) = 3.90, *p* = 0.058, η_p_^2^ = 0.12. The interaction of Group × Condition × Task was not significant, *F*(1, 30) = 0.06, *p* = 0.804, η_p_^2^ < 0.01. The interaction of Group × Condition × Feedback was not significant, *F*(1, 30) = 0.19, *p* = 0.667, η_p_^2^ = 0.01. The interaction of Group × Task × Feedback was not significant, *F*(1, 30) = 3.00, *p* = 0.093, η_p_^2^ = 0.09. The interaction of Condition × Task × Feedback was not significant, *F*(1, 30) = 0.04, *p* = 0.835, η_p_^2^ < 0.01. The interaction of Group × Condition × Task × Feedback was not significant, *F*(1, 30) = 1.16, *p* = 0.291, η_p_^2^ = 0.04. The findings about N1 are illustrated in Figure 3 and Figure 4 and Table 1.

### 3.2. FRN (210–280 ms, Fz)

There was a significant main effect of Feedback, *F*(1, 30) = 16.94, *p* < 0.001, η_p_^2^ = 0.36. Failure feedback evoked more negative FRN amplitudes than success feedback. However, the main effect of Group was not significant, *F*(1, 30) = 0.18, *p* = 0.675, η_p_^2^ = 0.01. The main effect of Condition was not significant, *F*(1, 30) = 0.80, *p* = 0.378, η_p_^2^ = 0.03, and the main effect of Task was not significant, *F*(1, 30) = 0.06, *p* = 0.812, η_p_^2^ < 0.01. 

**Figure 5:**
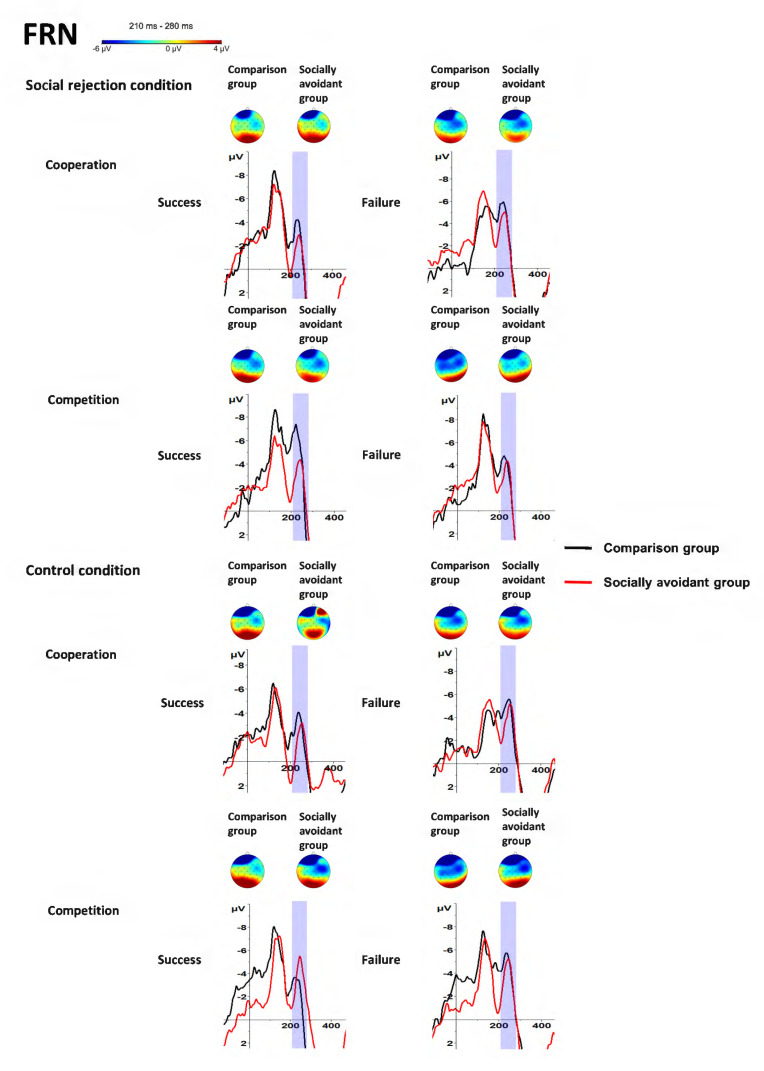
Grand-averaged FRN waveforms and scalp distributions in response to success and failure feedback in cooperative and competitive tasks in different conditions between socially avoidant group and comparison group.

The interaction of Task × Feedback was significant, *F*(1, 30) = 9.43, *p* = 0.005, η_p_^2^ = 0.24. Post hoc tests showed that failure feedback evoked more negative FRN amplitudes than success feedback in the cooperation task (*p* < 0.001) instead of the competition task (*p* = 0.251), and FRN amplitudes had no significant difference in the two tasks both for the success (*p* = 0.084) and failure feedback (*p* = 0.091). Importantly, the interaction of Group × Condition × Task × Feedback was significant, *F*(1, 30) = 4.58, *p* = 0.041, η_p_^2^ = 0.13. Post hoc tests showed that failure feedback evoked more negative FRN amplitudes than success feedback in the cooperation task of the control condition both in the socially avoidant (*p* = 0.010) and comparison group (*p* = 0.022) and in the cooperation task of the social rejection condition both in the socially avoidant (*p* = 0.019) and comparison group (*p* = 0.009). This feedback difference in FRN amplitude was also observed in the competition task of the control condition in the comparison group (*p* = 0.003) but not in the socially avoidant group (*p* = 0.991). Neither the socially avoidant (*p* = 0.485) nor the comparison group (*p* = 0.104) had a significant FRN amplitude difference in the competition task of the social rejection condition. However, there was no significant group difference in the cooperative success (*p* = 0.551) and failure (*p* = 0.626) and the competitive success (*p* = 0.210) and failure (*p* = 0.610) of the control condition, as well as in the cooperative success (*p* = 0.649) and failure (*p* = 0.477) and the competitive success (*p* = 0.233) and failure (*p* = 0.832) of the social rejection condition. Additionally, the control condition evoked more negative FRN amplitudes than the social rejection condition in the competitive failure of the comparison group (*p* = 0.039) instead of the socially avoidant group (*p* = 0.235). This condition difference was not observed in the competitive success of the socially avoidant (*p* = 0.184) and comparison group (*p* = 0.107), as well as in the cooperative success of the socially avoidant (*p* = 0.616) and comparison group (*p* = 0.498) and in the cooperative failure of the socially avoidant (*p* = 0.850) and comparison group (*p* = 0.884). Moreover, the cooperative task evoked more negative FRN amplitudes than the competitive task for the failure feedback in the social rejection condition in the comparison group (*p* = 0.020) instead of the socially avoidant group (*p* = 0.296). This task difference was not observed for the failure feedback in the control condition in the socially avoidant (*p* = 0.908) and comparison group (*p* = 0.866), as well as for the success feedback in the control condition in the socially avoidant (*p* = 0.063) and comparison group (*p* = 0.500) and for the success feedback in the social rejection condition in the socially avoidant (*p* = 0.330) and comparison group (*p* = 0.083). No other interaction was observed. The interaction of Group × Condition was not significant, *F*(1, 30) = 0.45, *p* = 0.508, η_p_^2^ = 0.02. The interaction of Group × Task was not significant, *F*(1, 30) = 0.85, *p* = 0.363, η_p_^2^ = 0.03. The interaction of Group × Feedback was not significant, *F*(1, 30) = 0.08, *p* = 0.785, η_p_^2^ < 0.01. The interaction of Condition × Task was not significant, *F*(1, 30) = 0.25, *p* = 0.618, η_p_^2^ = 0.01. The interaction of Condition × Feedback was not significant, *F*(1, 30) = 1.16, *p* = 0.291, η_p_^2^ = 0.04. The interaction of Group × Condition × Task was not significant, *F*(1, 30) = 0.88, *p* = 0.357, η_p_^2^ = 0.03. The interaction of Group × Condition × Feedback was not significant, *F*(1, 30) = 3.63, *p* = 0.067, η_p_^2^ = 0.11. The interaction of Group × Task × Feedback was not significant, *F*(1, 30) = 0.08, *p* = 0.776, η_p_^2^ < 0.01. The interaction of Condition × Task × Feedback was not significant, *F*(1, 30) = 3.78, *p* = 0.061, η_p_^2^ = 0.11. The findings about FRN are illustrated in Figure 3 and Figure 5 and Table 1.

### 3.3. P300 (300–410 ms, Fz)

There was a significant main effect of Condition, *F*(1, 30) = 22.27, *p* < 0.001, η_p_^2^ = 0.43. The social rejection condition evoked larger P300 amplitudes than the control condition, and the main effect of Task was significant, *F*(1, 30) = 15.46, *p* < 0.001, η_p_^2^ = 0.34. Competition evoked larger P300 amplitudes than cooperation. However, the main effect of Group was not significant, *F*(1, 30) = 1.28, *p* = 0.266, η_p_^2^ = 0.04, and the main effect of Feedback was not significant, *F*(1, 30) = 0.91, *p* = 0.347, η_p_^2^ = 0.03.

**Figure 6:**
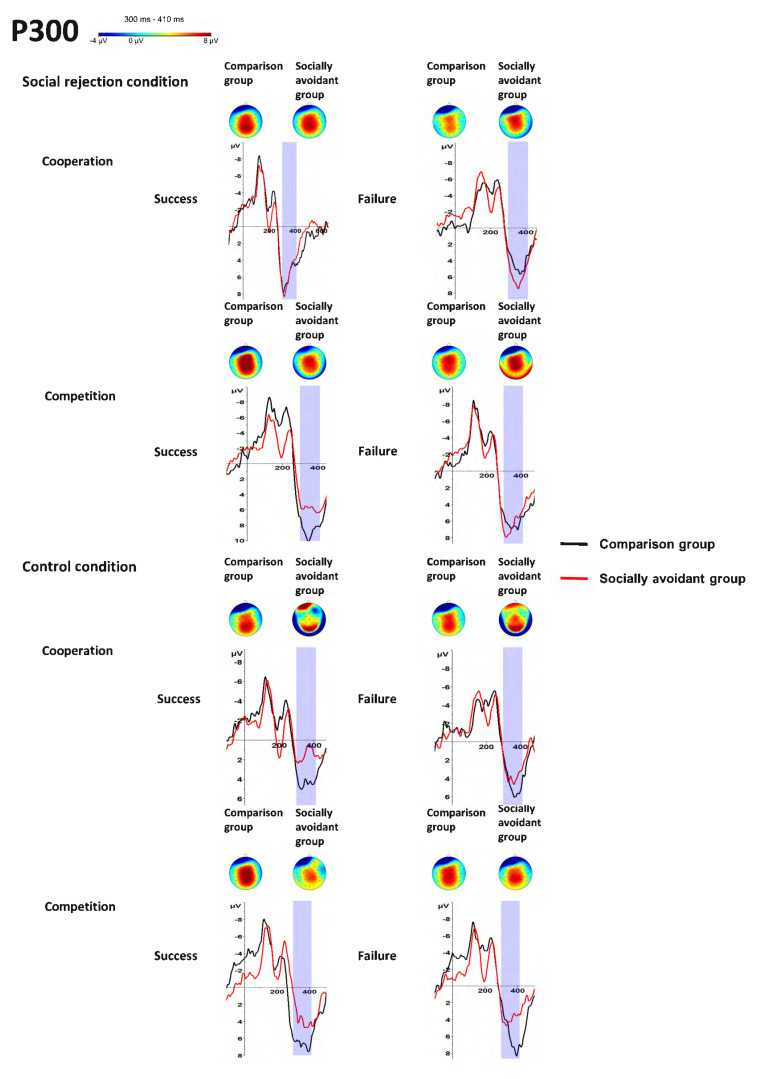
Grand-averaged P300 waveforms and scalp distributions in response to success and failure feedback in cooperative and competitive tasks in different conditions between socially avoidant group and comparison group.

The interaction of Group × Condition was significant, *F*(1, 30) = 5.24, *p* = 0.029, η_p_^2^ = 0.15. Post hoc tests showed that the social rejection condition evoked larger P300 amplitudes than the control condition only in the socially avoidant group (*p* < 0.001) instead of comparison group (*p* = 0.096), and the P300 amplitudes had no significant group difference in the social rejection (*p* = 0.790) and control condition (*p* = 0.061). The interactions of Condition × Feedback were significant, *F*(1, 30) = 7.91, *p* = 0.009, η_p_^2^ = 0.21. Post hoc tests showed that success feedback evoked larger P300 amplitudes than failure feedback only in the social rejection condition (*p* = 0.011) instead of the control condition (*p* = 0.559), and the social rejection condition evoked larger P300 amplitudes than the control condition both for the success (*p* < 0.001) and failure feedback (*p* = 0.012). The interactions of Task × Feedback were significant, *F*(1, 30) = 5.66, *p* = 0.024, η_p_^2^ = 0.16. Post hoc tests showed that success feedback evoked larger P300 amplitudes than failure feedback only in the competitive (*p* = 0.018) instead of the cooperative task (*p* = 0.535), and competition evoked larger P300 amplitudes than cooperation only for the success (*p* < 0.001) instead of the failure (*p* = 0.166) feedback. No other interaction was observed. The interaction of Group × Task was not significant, *F*(1, 30) = 3.29, *p* = 0.080, η_p_^2^ = 0.01. The interaction of Group × Feedback was not significant, *F*(1, 30) = 3.74, *p* = 0.062, η_p_^2^ = 0.11. The interaction of Condition × Task was not significant, *F*(1, 30) = 0.03, *p* = 0.874, η_p_^2^ < 0.01. The interaction of Group × Condition × Task was not significant, *F*(1, 30) = 1.30, *p* = 0.263, η_p_^2^ = 0.04. The interaction of Group × Condition × Feedback was not significant, *F*(1, 30) = 0.10, *p* = 0.751, η_p_^2^ < 0.01. The interaction of Group × Task × Feedback was not significant, *F*(1, 30) = 0.21, *p* = 0.649, η_p_^2^ = 0.01. The interaction of Condition × Task × Feedback was not significant, *F*(1, 30) = 0.92, *p* = 0.346, η_p_^2^ = 0.03. The interaction of Group × Condition × Task × Feedback was not significant, *F*(1, 30) = 0.82, *p* = 0.372, η_p_^2^ = 0.03. The findings about P300 are illustrated in Figure 3 and Figure 6 and Table 1.

### 3.4. RewP (250–350 ms, Cz)

There was a significant main effect of Group, *F*(1, 30) = 4.59, *p* = 0.040, η_p_^2^ = 0.13. The comparison group had larger RewP amplitudes than the socially avoidant group. There was a significant main effect of Condition, *F*(1, 30) = 5.00, *p* = 0.033, η_p_^2^ = 0.14. The social rejection condition evoked larger RewP amplitudes than the control condition. The main effect of Task was not significant, *F*(1, 30) = 0.04, *p* = 0.843, η_p_^2^ < 0.01.

**Figure 7:**
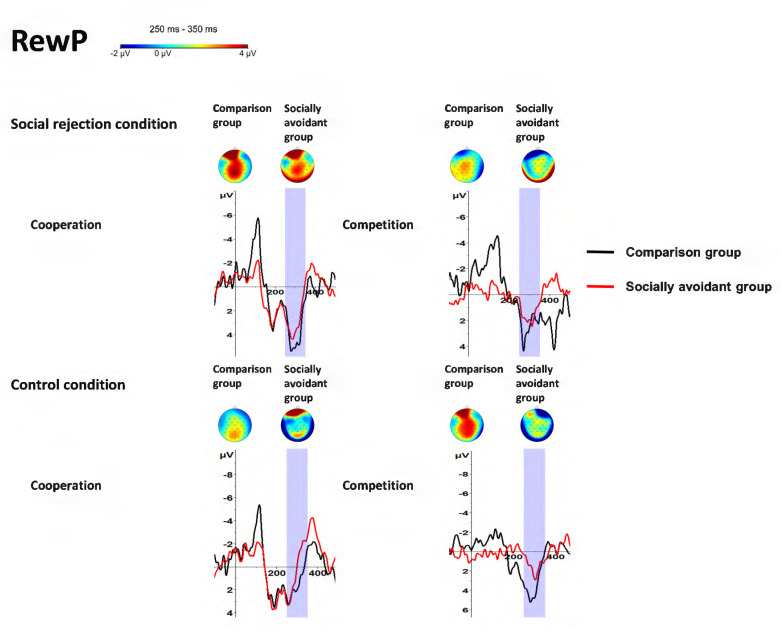
Grand-averaged RewP waveforms and scalp distributions in response to cooperative and competitive tasks in different conditions between socially avoidant group and comparison group.

The interaction of Condition × Task was significant, *F*(1, 30) = 9.27, *p* = 0.005, η_p_^2^ = 0.24. Post hoc tests showed that the social rejection condition evoked larger RewP amplitudes than the control condition only in cooperation (*p* < 0.001) instead of competition (*p* = 0.597), and cooperation evoked larger RewP amplitudes than competition in the social rejection condition (*p* = 0.041), while competition evoked larger RewP amplitudes than cooperation in the control condition (*p* = 0.046). No other interaction was observed. The interaction of Group × Condition was not significant, *F*(1, 30) = 0.64, *p* = 0.429, η_p_^2^ = 0.02. The interaction of Group × Task was not significant, *F*(1, 30) = 0.08, *p* = 0.786, η_p_^2^ < 0.01. The interaction of Group × Condition × Task was not significant, *F*(1, 30) = 0.09, *p* = 0.770, η_p_^2^ < 0.01. The findings about RewP are illustrated in Figure 3 and Figure 7 and Table 1.

## 4. Discussion

The present study investigated how socially avoidant emerging adults process social interaction feedback after undergoing social rejection by using computer game tasks combined with ERP recordings. The results indicated that socially avoidant individuals showed a different cognitive process from comparison individuals in the situation of human-to-human interaction. Specifically, socially avoidant emerging adults had lower RewP than comparison emerging adults to social feedback, and socially avoidant emerging adults had larger P300 amplitudes after social rejection, whereas comparison emerging adults did not. Moreover, socially avoidant and comparison emerging adults both had more negative N1 amplitudes in the social rejection condition than in the control condition (without social rejection). These results contribute to the understanding of the neural underpinnings of social avoidance that have important implications for interpersonal interaction in emerging adults [53,54]. 

### 4.1. Insensitivity towards Positive Social Feedback of Socially Avoidant Individuals

First, socially avoidant emerging adults had lower RewP activation to feedback information than comparison emerging adults during interpersonal interaction in both the control condition and social rejection condition, which supports Hypothesis 4. Previous research also demonstrated that individual differences in the degree of reward sensitivity are reflected in the amplitudes of RewP [52]. Thus, socially avoidant adults’ lower RewP amplitudes in the current study may speak volumes for their blunted neural responses to positive feedback during interaction. It suggests that social avoidance might be negatively correlated with the sensitivity to positive social feedback in emerging adults, consistent with previous research findings [16,27,33].

### 4.2. High Sensitivity towards Social Rejection of Socially Avoidant Individuals

Additionally, compared with the control condition, larger P300 amplitudes in the social rejection condition were only observed in the socially avoidant group, which is partially consistent with Hypothesis 3. P300 amplitudes are closely related to the mobilization of emotional resources when processing social feedback [55]. One possible explanation is that negative emotion elicited by social rejection could be well-regulated by comparison emerging adults; however, socially avoidant emerging adults who are more sensitive to rejection may put more emotional resources into regulating the negative emotion in a short time [56]. More than that, another research demonstrated that individuals with high rejection sensitivity had enhanced P300 amplitudes compared with those with low rejection sensitivity, supporting the results of P300 in this study [26]. It draws attention to socially avoidant adults’ strategies of emotional regulation, which is another potential problem during their interpersonal interaction.

### 4.3. The Impact of Social Rejection

Consistent with Hypothesis 1, it was discovered that in both groups, the N1 amplitudes in the social rejection condition were more negative than those in the control condition. Previous research suggested that enhanced N1 amplitude denotes a higher initial focus on the feedback stimuli [57], which may imply that social rejection might motivate socially avoidant emerging adults to regain acceptance in the following interaction task, just like comparison emerging adults [28]. Moreover, we detected that in the comparison group, competitive social context elicited more N1 neural activation than cooperative context. This was in line with previous ERP findings in the context of cooperative and competitive games [58]. In contrast, we did not find this pattern in the socially avoidant group. It seemed that socially avoidant emerging adults distributed equal attentional resources to cooperation and competition, which suggests that they might consider cooperation and competition as analogous social interactions, possibly due to their lack of social skills [19].

### 4.4. The Emphasis on the Failure Feedback

Additionally, FRN has been found to be a marker of reward prediction error in social interaction contexts [59]. Compared with positive feedback, negative feedback is supposed to evoke more negative FRN amplitudes [60]. The results showed that the comparison group in the competitive task showed expected more negative FRN amplitudes when processing failure feedback than success feedback in the control condition. Surprisingly, we did not find this pattern in the social rejection condition. A possible explanation is that they did not strongly expect the feedback in the competitive task after being rejected due to the need to bond with others [61]. In contrast, neither in the control condition nor the social rejection condition did socially avoidant emerging adults in the competitive task exhibit more negative FRN amplitudes when processing failure feedback than success feedback. It seemed that they did not pay much attention to failure feedback during the competitive task due to the fear of negative evaluation [62]. Nevertheless, in the cooperative task, both socially avoidant and comparison emerging adults had more negative FRN amplitudes for processing failure feedback than success feedback in the control condition and social rejection condition. That is, the two groups were both sensitive to the failure feedback in the cooperative task. This is probably because they cared about the cooperation as this is an important way to build relationships with others [63,64].

### 4.5. Research Contributions

In recent years, social neuroscience research has chosen cooperation and competition as two of the representative activities of interpersonal interaction [65,66]. In the current study, we used cooperative and competitive game tasks to examine the differences in social feedback processing between a socially avoidant group and a comparison group under the social rejection condition during actual interpersonal interaction. Cooperative and competitive tasks can produce social interaction feedback (i.e., success or failure), which is appropriate and has been validated to simulate real-life social interaction between two emerging adults [50,67]. In this study, the participant dyads were required to act as simultaneously as possible to win together in the cooperation task and act as fast as possible to beat their partner in the competition task. The specified goals of the game tasks may make it easy for socially avoidant individuals to interact with others, compared with the uncomfortable face-to-face talking interaction [23]. Thus, one of the study’s contributions could be the application of engaging interpersonal interaction tasks reflecting human social nature. It provides more information about socially avoidant individuals’ social interaction in a realistic setting than questionnaire measurement or single-participant neuroscience measurement [68]. 

The findings of the present study hold theoretical and practical implications. In terms of social avoidance theory, the results of the N1 and P300 amplitudes in the present study demonstrated that socially avoidant emerging adults may care about others’ acceptance despite their low approach motivation [4]. From a practical perspective, we found that lower RewP amplitudes may be a neural signature of socially avoidant emerging adults, which may provide a scientific basis for clinical screening of social avoidant individuals [33].

### 4.6. Limitations and Future Directions

It is important to keep the study’s limitations in mind when interpreting the results. Of note is that social avoidance is a relatively rare occurrence in the general population [11,12]. Although we had 430 participants fill in the social preference scale, only 17 of the large sample genuinely fitted the criterion for social avoidance, and the participants were university students from a single region in the present study. In the future study, we should increase the sample size and include participants from different cultural or social backgrounds to improve the generalizability of our findings [69]. Additionally, in the present study, we mainly examined the differences in the neural responses to the feedback in computer game tasks after social rejection between socially avoidant and comparison emerging adults. However, the neural synchrony and interaction between interacting individuals has received increasing interest among researchers in recent years [70]. Therefore, it is instructive and potentially instrumental for future studies to explore the interpersonal and neural interaction among socially avoidant emerging adults in light of the present research trend in social neuroscience [67]. In addition, future research could investigate the effects of more types of social feedback (e.g., personal feedback and non-personal feedback) on socially avoidant individuals [71]. Moreover, children and adolescents have a high prevalence of clinical social avoidance, indicating a significant developmental risk prior to emerging adulthood [72]. In the area of education, future research could explore social avoidance among children and adolescents to provide a developmental perspective and develop corresponding intervention programs (e.g., exercise intervention, mindfulness intervention) in families or school to promote their social development [73,74]. Last but not least, the low spatial resolution of the ERP technique constrained the interpretation of results concerning functional brain areas [75]. Future research could use fMRI (functional magnetic resonance imaging) and fNIRS (functional near-infrared spectroscopy) techniques to investigate social avoidance, providing more expansive mechanistic information [76].

## 5. Conclusions

Despite these limitations, this is the first study to explore socially avoidant emerging adults’ neural responses to social feedback information after social rejection in real interpersonal interaction. To sum up, when socially avoidant emerging adults received social interaction feedback, they displayed (a) more negative N1 amplitudes after social rejection; (b) more negative FRN amplitudes when processing failure feedback than success feedback in cooperation while not in competition; (c) higher P300 amplitudes after being socially rejected than not being socially rejected; and (d) lower RewP amplitudes than comparison emerging adults. In other words, socially avoidant emerging adults may exert more attentional and emotional resources to social feedback after social rejection [55,57]. Moreover, they might not expect negative social feedback in cooperation tasks [60]. Most importantly, they may have flaws in their reward sensitivity and emotional regulation during interpersonal interaction [52,56]. As a result, not only does this study provide a theoretical basis for explaining why social avoidance leads to social maladjustment and psychopathological risks, but it also provides an empirical basis for comprehending the neural underpinning of social avoidance.

## Figures and Tables

**Table 1:** Average amplitudes of different components between socially avoidant and comparison groups in different conditions and feedbacks.

Component	Condition	Feedback	Socially Avoidant Group (*n* = 16)		Comparison Group (*n* = 16)		t	*p*	d	95% CI	
			*M*	*SD*	*M*	*SD*					
N1	Social rejection	Cooperative success	−5.93	3.29	−6.73	1.67	0.87	0.392	0.31	−1.08	2.69
		Cooperative failure	−5.37	3.30	−4.20	2.68	−1.10	0.281	0.39	−3.34	1.00
		Competitive success	−6.24	2.81	−7.03	2.79	0.80	0.431	0.28	−1.23	2.81
		Competitive failure	−5.17	2.81	−6.51	2.85	1.35	0.189	0.48	−0.70	3.39
	Control	Cooperative success	−4.55	3.30	−4.66	1.70	0.12	0.903	0.04	−1.81	2.04
		Cooperative failure	−4.25	2.93	−3.15	2.40	−1.17	0.251	0.41	−3.04	0.82
		Competitive success	−5.36	5.01	−6.29	2.15	0.68	0.505	0.24	−1.92	3.77
		Competitive failure	−5.85	3.34	−5.92	2.42	0.07	0.943	0.03	−2.03	2.18
FRN	Social rejection	Cooperative success	−1.13	3.53	−1.80	4.65	0.46	0.649	0.16	−2.31	3.65
		Cooperative failure	−3.49	4.25	−4.43	3.03	0.72	0.477	0.25	−1.73	3.60
		Competitive success	−2.10	3.15	−3.56	3.60	1.22	0.233	0.43	−0.99	3.90
		Competitive failure	−2.60	3.40	−2.37	2.52	−0.21	0.832	0.08	−2.39	1.93
	Control	Cooperative success	−1.61	3.62	−2.45	4.24	0.60	0.551	0.21	−2.01	3.69
		Cooperative failure	−3.69	3.58	−4.28	3.21	0.49	0.626	0.17	−1.86	3.05
		Competitive success	−3.58	4.52	−1.75	3.47	−1.28	0.210	0.45	−4.74	1.08
		Competitive failure	−3.59	4.01	−4.14	1.37	0.52	0.613	0.18	−1.68	2.77
P300	Social rejection	Cooperative success	5.81	4.19	5.82	3.45	−0.01	0.990	0.01	−2.79	2.75
		Cooperative failure	5.97	5.00	4.52	3.08	0.99	0.333	0.35	−1.57	4.47
		Competitive success	6.47	3.68	8.75	3.91	−1.70	0.099	0.60	−5.03	0.46
		Competitive failure	5.87	4.63	6.34	4.79	−0.29	0.777	0.10	−3.88	2.93
	Control	Cooperative success	1.46	3.56	4.44	2.76	−2.64	0.013	0.93	−5.27	−0.67
		Cooperative failure	3.73	4.90	4.65	3.94	−0.59	0.560	0.21	−4.14	2.28
		Competitive success	3.97	3.97	6.69	3.25	−2.12	0.043	0.75	−5.34	−0.10
		Competitive failure	3.643	4.71	5.74	3.15	−1.48	0.149	0.52	−4.99	0.79
RewP	Social rejection	Cooperation	2.98	2.37	3.83	2.16	−1.06	0.298	0.37	−2.48	0.79
		Competition	1.60	2.83	2.45	2.87	−0.84	0.410	0.30	−2.90	1.22
	Control	Cooperation	0.20	2.96	1.52	2.89	−1.28	0.210	0.45	−3.43	0.79
		Competition	1.50	2.85	3.39	3.94	−1.56	0.131	0.55	−4.37	0.59

## Data Availability

The raw data supporting the conclusions of this article will be made available by the authors on request.

## Appendix A

In terms of behavioral data, RTs were examined by a 2 (Group: socially avoidant group vs. comparison group) × 2 (Condition: social rejection vs. control) × 2 (Task: cooperation vs. competition) × 2 (Feedback: success vs. failure) repeated measures ANOVA. The successful rate was an index of performance in the computer game tasks, which was computed as the number of cooperative/competitive success trials divided by the total number of cooperative/competitive trials in the cooperative/competitive task. The successful rate was examined by a 2 (Group: socially avoidant group vs. comparison group) × 2 (Condition: social rejection vs. control) × 2 (Task: cooperation vs. competition) repeated measures ANOVA. The results of the behavioral data recorded during the computer game tasks are as follows:

### Appendix A.1. Behavioral Results

#### Appendix A.1.1. RTs

The ANOVA revealed a significant main effect of Task, *F*(1, 30) = 15.29, *p* < 0.001, η_p_^2^ = 0.34, indicating that RTs in the cooperative task were longer than RTs in the competitive task, and there was a significant main effect of Feedback, *F*(1, 30) = 39.86, *p* < 0.001, η_p_^2^ = 0.57, indicating that RTs for the failure feedback were longer than RTs for the success feedback. The main effect of Group was not significant, *F*(1, 30) = 0.01, *p* = 0.938, η_p_^2^ < 0.01. The main effect of Condition was also not significant, *F*(1, 30) = 1.35, *p* = 0.255, η_p_^2^ = 0.04.

The interactions of Task × Feedback were significant, *F*(1, 30) = 28.92, *p* < 0.001, η_p_^2^ = 0.49. Post hoc tests suggest that RTs were longer in the cooperative task than in the competitive task both for the success (*p* < 0.001) and failure feedback (*p* = 0.043), and RTs were longer for the failure feedback than for the success feedback both in the cooperative (*p* = 0.014) and competitive task (*p* < 0.001). The interactions of Condition × Task × Feedback were significant, *F*(1, 30) = 5.65, *p* = 0.024, η_p_^2^ = 0.16. Post hoc tests showed that RTs were longer in cooperative success than in competitive success both in the social rejection (*p* < 0.001) and control condition (*p* < 0.001), while this effect was not significant for the failure feedback both in the social rejection (*p* = 0.066) and control condition (*p* = 0.082). RTs were longer for the failure feedback than for the success feedback in cooperation of the control condition (*p* = 0.008) and in competition of the social rejection (*p* < 0.001) and control condition (*p* < 0.001), but not in cooperation of the social rejection condition (*p* = 0.410). RTs were longer in the social rejection condition than in the control condition only in cooperative success (*p* = 0.010) instead of cooperative failure (*p* = 0.732), competitive success (*p* = 0.630), and failure (*p* = 0.924). No other interaction was observed. The interaction of Group × Condition was not significant, *F*(1, 30) < 0.01, *p* = 0.976, η_p_^2^ < 0.01. The interaction of Condition × Task was not significant, *F*(1, 30) = 2.88, *p* = 0.100, η_p_^2^ = 0.09. The interaction of Condition × Feedback was not significant, *F*(1, 30) = 1.35, *p* = 0.255, η_p_^2^ = 0.04. The interaction of Group × Task was not significant, *F*(1, 30) = 1.01, *p* = 0.324, η_p_^2^ = 0.03. The interaction of Group × Feedback was not significant, *F*(1, 30) = 0.46, *p* = 0.504, η_p_^2^ = 0.02. The interaction of Group × Condition × Task was not significant, *F*(1, 30) = 0.59, *p* = 0.450, η_p_^2^ = 0.02. The interaction of Group × Condition × Feedback was not significant, *F*(1, 30) = 0.19, *p* = 0.667, η_p_^2^ = 0.01. The interaction of Group × Task × Feedback was not significant, *F*(1, 30) = 1.49, *p* = 0.231, η_p_^2^ = 0.05. The interaction of Group × Condition × Task × Feedback was not significant, *F*(1, 30) = 0.44, *p* = 0.513, η_p_^2^ = 0.01.

#### Appendix A.1.2. Successful Rate

The ANOVA revealed no significant main effect. The main effect of Group was not significant, *F*(1, 30) = 3.20, *p* = 0.084, η_p_^2^ = 0.10. The main effect of Condition was not significant, *F*(1, 30) = 2.80, *p* = 0.104, η_p_^2^ = 0.09. The main effect of Task was not significant, *F*(1, 30) = 3.68, *p* = 0.065, η_p_^2^ = 0.11. Moreover, no interaction was observed. The interaction of Group × Condition was not significant, *F*(1, 30) = 2.27, *p* = 0.143, η_p_^2^ = 0.07. The interaction of Group × Task was not significant, *F*(1, 30) = 2.91, *p* = 0.099, η_p_^2^ = 0.09. The interaction of Condition × Task was not significant, *F*(1, 30) = 2.61, *p* = 0.117, η_p_^2^ = 0.08. The interaction of Group × Condition × Task was not significant, *F*(1, 30) = 1.86, *p* = 0.183, η_p_^2^ = 0.06.

**Table A1:** Average RTs and successful rate between socially avoidant group and comparison group.

	Condition	Task	Feedback	Socially Avoidant Group (*n* = 16)		Comparison Group (*n* = 16)		t	*p*	d	95% CI	
				*M*	*SD*	*M*	*SD*					
Average RTs (ms)	Social rejection	Cooperation	Success	341.76	139.71	364.80	153.10	−0.45	0.660	0.16	−128.87	82.78
			Failure	351.81	95.98	376.90	109.07	−0.69	0.495	0.24	−99.27	49.09
		Competition	Success	218.18	45.37	211.84	32.75	0.45	0.654	0.16	−22.23	34.91
			Failure	345.73	139.03	296.10	46.86	1.35	0.186	0.48	−25.28	124.53
	Control	Cooperation	Success	301.52	102.84	312.46	108.50	−0.29	0.772	0.10	−87.27	65.38
			Failure	350.12	99.04	363.49	144.44	−0.31	0.762	0.11	−102.78	76.05
		Competition	Success	221.32	27.53	215.87	30.78	0.53	0.601	0.19	−15.63	26.54
			Failure	331.37	89.19	307.44	85.09	0.78	0.443	0.27	−39.00	86.88
Successful rate (%)	Social rejection	Cooperation		52.81	17.31	52.53	17.44	0.05	0.965	0.02	−12.27	12.82
		Competition		57.99	12.27	42.55	12.64	3.51	0.001	1.24	6.45	24.43
	Control	Cooperation		58.94	11.77	59.15	11.30	−0.05	0.959	0.02	−8.54	8.12
		Competition		52.52	12.67	48.19	12.88	0.96	0.346	0.34	−4.90	13.55

## Appendix B

To eliminate the possible impact of correlative variables on the dyads’ social interaction, we examined age, gender, and social anxiety once in the first visit of this experiment. Emotional states, self-efficacy, self-esteem, basic psychological needs, and aggression were examined separately both in the social rejection and control conditions. Repeated measures ANOVAs of N1, FRN, P300, and RewP components were employed and the above-mentioned correlative variables were set as covariates separately.

The scales used to examine correlative variables are described as follows:

*Social anxiety*: The 10-item Social Anxiety Scale (SASC) [77] was used to assess the subjective anxiety and fear from social behavior (Cronbach’s α = 0.77). The scale employed a 3-level rating (0 = *Never*, 1 = *Sometimes*, 2 = *Always*).

*Emotional states*: The emotional states were assessed by the 20-item Positive and Negative Affect scale (PANAS) [78] on a 5-point scale (1 = *Not at all* to 5 = *A lot*). Ten items assessed positive emotional state and another 10 items assessed negative emotional state. In the current study, Cronbach’s α of the PANAS was 0.91 in the control condition and 0.92 in the social rejection condition.

*Self-efficacy*: The participants’ self-efficacy was assessed by the 10-item General Self-efficacy Scale (GSES) [79] on a 4-point scale (1 = *Not at all* to 4 = *A lot*). In this study, Cronbach’s α of the scale was 0.85 in the control condition and 0.88 in the social rejection condition.

*Self-esteem*: The 10-item Self-esteem Scale (SES) was used to assess the self-esteem of participants [65] on a 4-point scale (1 = *Not at all* to 4 = *A lot*). In this study, Cronbach’s α of the scale was 0.90 in the control condition and 0.83 in the social rejection condition.

*Basic psychological needs*: The 19-item Basic Psychological Needs Scale (in Chinese) was adopted to assess the extent of basic psychological needs of participants [66] on a 7-point scale (1 = *Not at all* to 7 = *A lot*). In this study, Cronbach’s α of the scale was 0.83 in the control condition and 0.83 in the social rejection condition.

*Aggression*: A 30-item Chinese version of the Buss and Perry Aggression Questionnaire was used to assess the aggression of the participants [67] on a 5-point scale (1 = *Not at all* to 5 = *A lot*). In this study, Cronbach’s α of the scale was 0.84 in the control condition and 0.87 in the social rejection condition.

The results of covariate analysis are as follows:

N1 Component

Age: *F*(1, 29) = 0.08, *p* = 0.784, η_p_^2^ < 0.01; gender: *F*(1, 29) = 0.38, *p* = 0.541, η_p_^2^ = 0.01; social anxiety: *F*(1, 29) = 1.83, *p* = 0.187, η_p_^2^ = 0.06; positive emotional state in the control condition: *F*(1, 29) = 0.41, *p* = 0.528, η_p_^2^ = 0.01; positive emotional state in the social rejection condition: *F*(1, 29) = 0.06, *p* = 0.809, η_p_^2^ < 0.01; negative emotional state in the control condition: *F*(1, 29) = 1.83, *p* = 0.186, η_p_^2^ = 0.06; negative emotional state in the social rejection condition: *F*(1, 29) = 0.10, *p* = 0.754, η_p_^2^ < 0.01; self-efficacy in the control condition: *F*(1, 29) = 0.18, *p* = 0.679, η_p_^2^ = 0.01; self-efficacy in the social rejection condition: *F*(1, 29) = 0.40, *p* = 0.531, η_p_^2^ = 0.01; self-esteem in the control condition: *F*(1, 29) = 0.27, *p* = 0.606, η_p_^2^ = 0.01; self-esteem in the social rejection condition: *F*(1, 29) = 0.50, *p* = 0.486, η_p_^2^ = 0.02; basic psychological needs in the control condition: *F*(1, 29) = 1.99, *p* = 0.169, η_p_^2^ = 0.06; basic psychological needs in the social rejection condition: *F*(1, 29) = 2.59, *p* = 0.118, η_p_^2^ = 0.08; aggression in the control condition: *F*(1, 29) = 0.09, *p* = 0.771, η_p_^2^ < 0.01; and aggression in the social rejection condition: *F*(1, 29) = 0.05, *p* = 0.825, η_p_^2^ < 0.01.

FRN Component

Age: *F*(1, 29) = 0.32, *p* = 0.576, η_p_^2^ = 0.01; gender: *F*(1, 29) = 0.50, *p* = 0.484, η_p_^2^ = 0.02; social anxiety: *F*(1, 29) = 11.80, *p* = 0.002, η_p_^2^ = 0.29; positive emotional state in the control condition: *F*(1, 29) = 10.53, *p* = 0.003, η_p_^2^ = 0.27; positive emotional state in the social rejection condition: *F*(1, 29) = 11.88, *p* = 0.002, η_p_^2^ = 0.29; negative emotional state in the control condition: *F*(1, 29) = 0.16, *p* = 0.693, η_p_^2^ = 0.01; negative emotional state in the social rejection condition: *F*(1, 29) = 9.16, *p* = 0.005, η_p_^2^ = 0.24; self-efficacy in the control condition: *F*(1, 29) = 0.24, *p* = 0.630, η_p_^2^ = 0.01; self-efficacy in the social rejection condition: *F*(1, 29) = 0.10, *p* = 0.757, η_p_^2^ < 0.01; self-esteem in the control condition: *F*(1, 29) < 0.01, *p* = 0.985, η_p_^2^ < 0.01; self-esteem in the social rejection condition: *F*(1, 29) = 0.28, *p* = 0.602, η_p_^2^ = 0.01; basic psychological needs in the control condition: *F*(1, 29) = 0.65, *p* = 0.428, η_p_^2^ = 0.02; basic psychological needs in the social rejection condition: *F*(1, 29) = 1.62, *p* = 0.213, η_p_^2^ = 0.05; aggression in the control condition: *F*(1, 29) = 1.47, *p* = 0.235, η_p_^2^ = 0.05; and aggression in the social rejection condition: *F*(1, 29) = 3.52, *p* = 0.071, η_p_^2^ = 0.11.

P300 Component

Age: *F*(1, 29) = 0.21, *p* = 0.650, η_p_^2^ = 0.01; gender: *F*(1, 29) = 1.49, *p* = 0.232, η_p_^2^ = 0.05; social anxiety: *F*(1, 29) = 0.54, *p* = 0.468, η_p_^2^ = 0.02; positive emotional state in the control condition: *F*(1, 29) = 0.64, *p* = 0.432, η_p_^2^ = 0.02; positive emotional state in the social rejection condition: *F*(1, 29) = 0.12, *p* = 0.730, η_p_^2^ < 0.01; negative emotional state in the control condition: *F*(1, 29) = 0.05, *p* = 0.824, η_p_^2^ < 0.01; negative emotional state in the social rejection condition: *F*(1, 29) = 3.30, *p* = 0.080, η_p_^2^ = 0.10; self-efficacy in the control condition: *F*(1, 29) = 0.30, *p* = 0.586, η_p_^2^ = 0.01; self-efficacy in the social rejection condition: *F*(1, 29) = 0.99, *p* = 0.329, η_p_^2^ = 0.03; self-esteem in the control condition: *F*(1, 29) = 1.68, *p* = 0.205, η_p_^2^ = 0.06; self-esteem in the social rejection condition: *F*(1, 29) = 2.06, *p* = 0.162, η_p_^2^ = 0.07; basic psychological needs in the control condition: *F*(1, 29) = 0.19, *p* = 0.664, η_p_^2^ = 0.01; basic psychological needs in the social rejection condition: *F*(1, 29) = 0.10, *p* = 0.756, η_p_^2^ < 0.01; aggression in the control condition: *F*(1, 29) = 1.62, *p* = 0.213, η_p_^2^ = 0.05; and aggression in the social rejection condition: *F*(1, 29) = 3.39, *p* = 0.076, η_p_^2^ = 0.11.

RewP Component

Age: *F*(1, 29) = 0.29, *p* = 0.597, η_p_^2^ = 0.01; gender: *F*(1, 29) = 0.01, *p* = 0.908, η_p_^2^ < 0.01; social anxiety: *F*(1, 29) = 0.15, *p* = 0.699, η_p_^2^ = 0.01; positive emotional state in the control condition: *F*(1, 29) = 0.60, *p* = 0.446, η_p_^2^ = 0.02; positive emotional state in the social rejection condition: *F*(1, 29) = 0.18, *p* = 0.676, η_p_^2^ = 0.01; negative emotional state in the control condition: *F*(1, 29) = 0.39, *p* = 0.539, η_p_^2^ = 0.01; negative emotional state in the social rejection condition: *F*(1, 29) = 0.50, *p* = 0.484, η_p_^2^ = 0.02; self-efficacy in the control condition: *F*(1, 29) = 0.02, *p* = 0.892, η_p_^2^ < 0.01; self-efficacy in the social rejection condition: *F*(1, 29) = 0.02, *p* = 0.879, η_p_^2^ < 0.01; self-esteem in the control condition: *F*(1, 29) = 0.68, *p* = 0.417, η_p_^2^ = 0.02; self-esteem in the social rejection condition: *F*(1, 29) = 0.27, *p* = 0.607, η_p_^2^ = 0.01; basic psychological needs in the control condition: *F*(1, 29) = 0.21, *p* = 0.649, η_p_^2^ = 0.01; basic psychological needs in the social rejection condition: *F*(1, 29) = 0.08, *p* = 0.778, η_p_^2^ < 0.01; aggression in the control condition: *F*(1, 29) = 3.30, *p* = 0.080, η_p_^2^ = 0.10; and aggression in the social rejection condition: *F*(1, 29) = 1.86, *p* = 0.183, η_p_^2^ = 0.06.
